# Environmental insults and compensative responses: when microbiome meets cancer

**DOI:** 10.1007/s12672-023-00745-9

**Published:** 2023-07-15

**Authors:** Sunil Nagpal, Sharmila S. Mande

**Affiliations:** 1 TCS Research, Tata Consultancy Services Ltd, Pune, 411013 India; 2 CSIR-Institute of Genomics and Integrative Biology (CSIR-IGIB), New Delhi, 110025 India; 3 Academy of Scientific and Innovative Research (AcSIR), Ghaziabad, 201002 India

## Abstract

Tumor microenvironment has recently been ascribed a new hallmark—the polymorphic microbiome. Accumulating evidence regarding the tissue specific territories of tumor-microbiome have opened new and interesting avenues. A pertinent question is regarding the functional consequence of the interface between host-microbiome and cancer. Given microbial communities have predominantly been explored through an ecological perspective, it is important that the foundational aspects of ecological stress and the fight to ‘survive and thrive’ are accounted for tumor-micro(b)environment as well. Building on existing evidence and classical microbial ecology, here we attempt to characterize the ecological stresses and the compensative responses of the microorganisms inside the tumor microenvironment. What insults would microbes experience inside the cancer jungle? How would they respond to these insults? How the interplay of stress and microbial quest for survival would influence the fate of tumor? This work asks these questions and tries to describe this underdiscussed ecological interface of the tumor and its microbiota. It is hoped that a larger scientific thought on the importance of microbial competition sensing vis-à-vis tumor-microenvironment would be stimulated.

## Introduction

Cancer represents a class of complex, treatment elusive, multimodal diseases typically characterized by uncontrolled cell division primarily attributed to oncogenic mutations [1–3]. An array of well-founded additional hallmarks like hyper-proliferative signalling, apoptosis-evasion, angiogenesis, hyper-anabolism, inflammation, invasive-metastasis and more have traditionally helped view the vast genotypic and phenotypic diversity of cancers through the lens of a unified concept of ‘Tumor-microenvironment (TME)’ [1, 3, 4]. TME, consisting of a heterogenous collection of normal cells, vasculature, immune cells, signalling/growth factors, metabolites, and extra-cellular matrix around the (transformed) cancer cells, is in fact an ecosystem in itself [3]. It has its own unique nutrient, acid and oxygen profiles, peculiar oxidative stress and distinct cellular (dis)organization [3, 5–7]. It is the dynamic and integrated interplay of various entities in the challenging conditions of TME, which is now understood to determine the fate of cancer towards growth, invasion, or suppression [1, 3, 8, 9]. Identification of the hallmarks of cancer has thus significantly aided integrative and organized research towards the factors that contribute to cancer development/ suppression [8]. Recently, a new hallmark has been ascribed to the complex ecosystem of TME—the host associated microbiome [4, 10]. Accumulating evidence have now consolidated the belief that the vast gene pool and communities of trillions of microorganisms (bacteria, fungi, viruses, protists) in the human body (collectively termed as the human microbiome), may, in-part, crosstalk with the other entities of the TME [4, 11, 12]. Given the compelling evidence of microbial (particularly bacterial) regulation of general host-immunity and physiological homeostasis [11, 12], rational questions are now being raised about the potential role that the host microbial communities can play inside the TME, especially towards cancer development and management.

Microbial association with oncopathology has in fact been discussed for decades, with reports of anti-cancerous activity of bacterial toxins dating back to a century ago [13]. Discovery of specific microorganisms inside various tumors and their causal associations have consistently been reported in the past [14, 15]. These include the oncogenic linkages of infection or colonization by (opportunistic) pathogens like *Helicobacter pylori* (gastric cancer), *Chlamydia trachomatis* (cervical cancer), *Salmonella typhi* (gall bladder cancer), *Chlamydophila pneumoniae* (lung cancer), *Streptococcus bovis* (colorectal cancer), *Fusobacterium nucleatum* (colon cancer), *Bacteroides fragilis* (colorectal cancer) etc. [10, 14, 16, 17]. However, recently a comprehensive characterization of the collective microbiome associated with different human tumor types was achieved at a large scale (amassing more than 1500 samples) [18]. Attracting significant attention to the tumor microbial ecology, it laid the foundation for potential tissue specific territories of tumor microbiome [18]. Furthermore, the breakthrough attempted to quash many prevailing doubts pertaining to the contamination linked discoveries through multiple negative controls and contaminant filtration strategies [18, 19]. Several reports characterizing the tumor associated microbiome have now emerged in the last 3 years, consolidating the existence and importance of the tumor micro(b)environment [18, 20–32]. An increasing number of comprehensive review reports, attempting to delineate the immuno-oncology-microbiome (IOM) axis, also emphasize the interest in understanding the functional significance of the microbial interface with cancer [10, 15]. Previously, reports of success in building an onco-diagnostic tool using tissue and blood associated microbial-signatures in treatment-naive cancer patients had also highlighted the under looked sparse microbial content of the tumors [33]. These seminal studies have now provided significant guiding evidence towards (i) primarily the differential microbial community compositions in and around cancer cells [18], (ii) a microbiota regulated onco-immune system [10] and (iii) a preference of the microbes to inhabit specific microniches in the TME [32]. Taken together, while onco-immunology has till date played a pivotal role in deciphering the functional aspects of cancer-microbiota crosstalk, the functional models for tumor associated ‘communities of microbes’ warrant further research. The foundational concepts of a rather closely tied discipline - Ecology, may here aid the functional integration of microbial community studies with oncology. Oncoecology, from the perspective of the tumor microbiome can in fact raise some pertinent and relevant questions:

- What challenges or insults would microbes face while transitioning to a dynamic, harsh, and complex environment of tumors?
- How would microbes respond to such challenges?
- What would be the collateral impact of microbial response to such insults, on the tumor/TME? Would it be anticancer or oncogenic?

In the current state of the art, the tumor-microbe interface remains underdiscussed from the above mentioned point of view of oncoecology. We attempt to address this gap by reviewing the environmental conditions of the TME that may act as ‘ecological stresses’ for the tumor associated microbiota. The foundational concepts of microbial response to environmental stresses are also reviewed to interject the plausible compensative responses of the tumor-microbiome against the perceived ecological insults. The synthesized knowledge subsequently helps in building perspectives on the collateral impacts of this environmental sensing in the tumor micro(b)environment.

## The tumor-microbe interface

Success of colonization of tumors by microbes is expected to depend primarily on two factors–

(i) an influx of the micro-organisms
(ii) availability of conducive conditions for them to survive, thrive and co-exist in the tumor microenvironment.

**Fig. 1:**
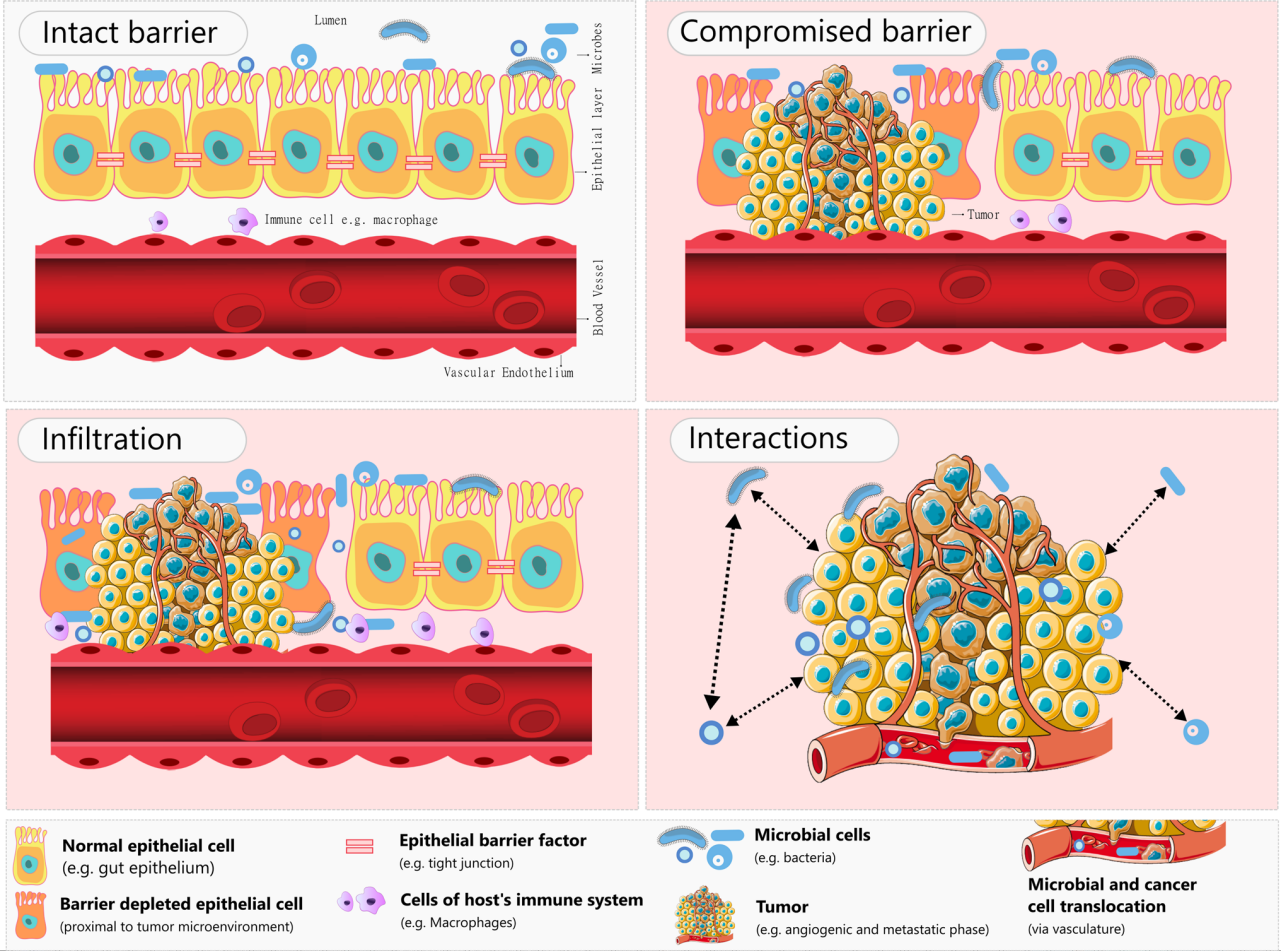
Graphical representation of a scenario, showcasing events that can contribute to intratumoral microbiome. Panel ‘intact barrier’ represents epithelial layer (with intact epithelial barrier depicted through tight junctions), host immune cells, luminal/mucosal microbiota and blood vessels in a healthy state. Panel ‘compromised barrier’ represents depletion or weakening of epithelial barrier due to tumor microenvironment, providing opportunity to the host microbiota to breach the barrier. Panel ‘infiltration’ depicts the event of microbial infiltration of compromised epithelial layer and consequent interface with tumor microenvironment (TME), including the associated host immune cells. Excess immune cells are also recruited to the TME and neighbouring tissues due to the infiltration. Panel ‘interactions’ represents the event of the host-microbiota entering the jungle of tumor micro-environment and commencement of its quest for surviving and thriving (represented by arrows showing microbe-tumor/microenvironment and microbe-microbe interactions in the ‘interactions’ panel)

While the influx can be driven by factors like luminal infiltrations (Fig. 1) through compromised epithelial/mucosal barrier [34, 35], inheritance from normal adjacent tissues or NAT [18], zipper/trigger mechanisms of bacterial invasion [36] and circulatory contributions from leaky vasculature of the tumor [37, 38], survival, thrival, and co-existence is not only dependent on the availability of favourable micro-niches in the tumor microenvironment [32] but also on the activation of microbial stress responses against the perceived unfavourable ‘environmental insults’ (including the inter/intraspecies competition).

## The environmental insults inside the tumor microenvironment

**Fig. 2:**
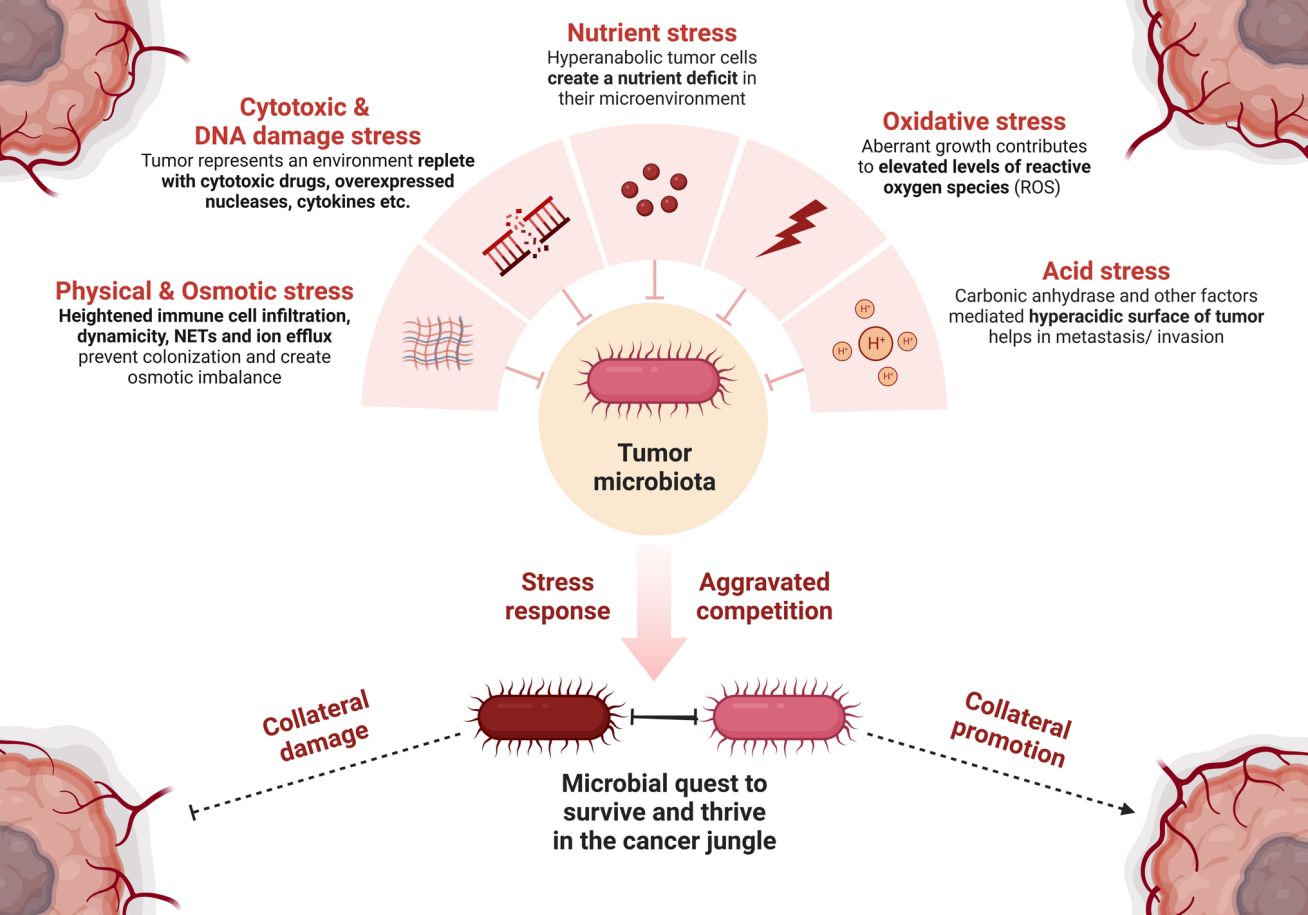
Characterization of the key environmental insults offered by tumor-microenvironment to the infiltrating/intratumoral microbes. Nutrient stress, oxidative stress, acid stress, physical & osmotic stress and DNA damaging/cytotoxic stress in combination are expected to offer significant and persistent insults to the incoming/prevailing microbes in the tumor microenvironment. Microbial response to these stresses may in collateral promote or damage the tumor cells

Tumor microenvironment, in fact, can offer several challenges/insults to the visiting microbes as summarised in the graphical abstract and the Fig. 2. These include:

### Nutrient stress

Two key hallmarks of tumor are the hyperproliferation and hyperanabolism [4]. The unregulated proliferation leads to heightened energy and anabolic needs [4, 39]. Consequently, the tumor-microenvironment is always nutrient deprived. While the adaptively programmed cancer cells are always hungry for glucose to utilize it ‘effectively and rapidly’ through the Warburg effect [39, 40], the oncogenic mutations generally lead to a heterogenous cancerous mass dependent on ‘not one but various limiting substrates’, leading to a continuous pressure on a variety of nutrients in the milieu of the tumor [39]. This is further aggravated in the Cancer stem cells (CSCs) which represent a subpopulation in the tumor microenvironment, and are undifferentiated and highly aggressive [41]. The infiltrating and intratumor microorganisms are therefore expected to encounter a perpetually hungry and aggressive competitor as soon as they enter the tumor-microenvironment. How the visitors (microbes) would respond to this nutrient stress, can potentially guide the development of meaningful functional models of the tumor-micro(b)environment. Notably, the necrotic regions in the tumor represent an exception, offering a less competitive, nutrient rich hypoxic microniche for the growth and proliferation of the microorganisms [32, 42].

### Oxidative stress

Reactive oxygen species (ROS), the free radicals, bearing unpaired reactive electron in their valence shells, are normal byproducts of cellular respiration (oxidative phosphorylation). Redox homeostasis is critical for maintaining a balance between the reactive oxygen species (ROS) and antioxidants [43]. The antioxidant-enzymes (e.g. superoxide dismutase or SOD) mediated redox balance prevents the normal cells from cytotoxic damage and checks the tumorigenic effects of ROS as well [43]. The balance of redox homeostasis however doesn’t prevail in the tumor microenvironment which is replete with the ROS (the oxidative stress) due to hyperproliferation, hyper-metabolism, mitochondrial dysfunction, infiltrating immune cells, genetic (oncogenic) alterations, upregulated oxidases, peroxisome activity and among more [5]. While primarily tumorigenic, ROS can inhibit tumors as well owing to their cytotoxic nature [5, 43]. Cancer cells therefore employ adaptive metabolic modes of managing the high ROS levels through NADPH accumulation, glutamine and folate metabolism etc. [5]. The incoming microorganisms would also need independent intrinsic mechanisms to fend this insult off or perish due to the deleterious effects of free radicals on various macromolecules (DNA, proteins, lipids, etc.), including an eventual cell death. The collateral impact of said adaptive mechanisms on the tumor (microenvironment) would be interesting to probe and understand.

### Physical and osmotic stress

Tumors are like wounds that never heal [44]. Unlike normal tissues with a stable structure, composition and biochemistry, tumor microenvironment is highly dynamic and unstable. This dynamicity is attributed to the continuous angiogenesis, leaky vasculature, plasma extravasation,  and a progression towards desmoplasia or solid tumors [44]. Furthermore, the compressive stress faced by solid tumors, while invading and navigating through the normal adjacent tissue, causes increased intracellular tonicity (osmotic pressure), triggering the upregulation of sodium efflux by tumors into the TME [7]. Consequently, tumoral microbes are expected to face significant (i) 'mechanical stress' due to the dynamic spatio-temporal composition of tumor, preventing surface attachment or promoting detachment, hence challenging the colonization of the TME and (ii) 'osmotic stress' due to the efflux of ions challenging microbial survival under the perturbed osmo-homeostasis. The continuous infiltration of inflammatory and immune cells [44, 45], including macrophages and neutrophils, in the never healing wounds of tumor, can further aggravate the physical stress on the microbes seeking a firm attachment or colonization. A notable example of immune surveillance mediated physical stress pertains to the expression of neutrophil extracellular traps (NETs) in the tumor microenvironment [46]. NETs are extracellular complexes containing fibres of decondensed chromatin (DNA), decorating protein granules, antimicrobial proteins and histones used as a self-sacrificing defence mechanism (NETosis) by the neutrophils to trap and kill invading microbial pathogens too large to engulf [47]. There are mixed evidence towards the impact of NETs on tumors. Studies have indicated an anti-cancer role of NETs through apoptosis, necrosis, ROS and H_2_O_2_ mediated cytotoxicity [46]. Evidence are also accumulating that tumors are more inclined to leverage the NETs for proliferation and micro-metastasis [48, 49]. It is however invariably well-founded that NETs function to inhibit or kill invading microbes. The strategies adopted by microbes to adapt against or address these environmental stresses interfering with colonization would therefore be additionally critical in understanding the microbe-tumor interplay, especially from a spatio-temporal standpoint.

### Acid stress

The Warburg-effect or the preference for glycolytic metabolism is known to lower the pH of tumor-microenvironment [40, 50]. This is attributed to the rapid extrusion of accumulated lactate to the extracellular environment. Additionally, the acidosis is also promoted by the membrane-bound carbonic anhydrases through the release of protons while sequestering carbon dioxide [50]. Both these acidification promoting mechanisms are essentially ‘adaptive responses’ of the cancer cells towards heightened energy needs (glycolytic metabolism) and hypoxia (over expressed carbonic anhydrases). As a result, tumor-microenvironment exhibits an inverted pH gradient (pH_extracellular_ < pH_intracellular_), opposite to the normal tissues/cellular environments, where extra-celluar pH is higher than the intracellular pH. An alkaline intracellular pH helps tumors to proliferate and evade apoptosis within the physiological pH range (7.2–7.4), while an acidic microenvironment (6.3–7.0) enables activation of proteases and metastatic pathways, enabling cellular dispersion, immune-evasion, drug-resistance, and invasion of healthy tissues [50]. Given the heterogenous nature of tumors, a stable pH gradient cannot be expected in the tumor-microenvironment. Moreover, the steepness in the pH changes between the normal cellular environment and the tumor-microenvironment can also be dictated by the anatomical geography of the host (e.g. normal extracellular pH in: airway mucosa ~ 5.5–7.9, stomach ~ 1.5–3.5, colon: 6.1–7.5) [51, 52]. It would be interesting to understand how the dynamic, slightly acidic pH environment of tumors can affect the survival of the infiltrating microbes, which can having diverse pH sensitivities. The acidosis driven dispersion/metastasis of cancer cells can additionally exert a physical stress on the existing colonies or the microbes seeking a site of attachment [50, 53]. Tumor-associated pH gradients and associated heterogeneity can therefore potentially influence colonization and subsequent interactions between the tumor and the microbiome, warranting further research.

### Xenobiotic and DNA damage stress

In addition to the intrinsic hallmarks of cancer offering a variety of stresses to the visiting microbiota, the extrinsic interventional regimens exert tremendous stress on the tumor, normal tissues, and the native microbiome in and outside the tumor-microenvironment. Cytotoxic and inhibitory effects of the xenobiotic chemotherapeutic agents on microbes, much of which are attributed to the DNA damaging traits of these chemicals, are infact well founded [54, 55]. Given that antibiotics have consistently been employed in many chemotherapies for their anti-cancer properties, the DNA damaging, inhibitory or microbicidal action of the chemotherapeutic regimens are rather expected [56]. Maier and colleagues however also demonstrated, in-vitro, the inhibitory effects of even the non-antibiotic chemotherapeutic agents on well-known commensal microorganisms of the human gut [57]. It has also been recently proven that the conventional myelosuppressive chemotherapy disrupts intestinal microbiome [58]. The heterogeneity added to the tumor-microenvironment by the (often) harsh therapeutic regimens, is therefore expected to add to the insults faced by the visiting microbes. Understanding the microbial response towards exposure to this stressful microenvironment replete with the chemotherapeutic agents can not only (potentially) describe the ecological basis of the consolidation of tumor-microbiome, but also the microbe-drug-tumor interplay.

Furthermore, microbial genetic material can also be stressed by the ROS (as described earlier) and the pool of nucleases expressed in the tumor-microenvironment. Nucleases, the enzymes that can hydrolyse nucleic acids, have consistently been perceived as promising biomarkers for cancer. This is attributed to their frequently observed overexpression, with some reports of interindividual variability, in the cancers of various types [59]. Nucleases however are also critical towards establishing innate immunity against bacteria and viruses. This is achieved through pattern recognition receptor (PRR) mediated pathways, which are aberrantly expressed in tumors [60]. These nucleic acid degraders, ranging from exonucleases to endonucleases, are known to be expressed intracellularly, extracellularly as well as ‘on the membrane’ of cancer cells, marking their omnipresence in the tumor-microenvironment [61]. While the functional significance of the largely overexpressed tumoral nucleases remain to be fully understood, studies have associated their overexpression with aggravated tumor growth and digressive response to chemotherapy [59]. Notably, nucleases can also have bacterial origin, predominantly employed in the bacterial warfare for survival in the competitive environments, targeting the non-self microbes and host cells. Regardless of the origin, the targeting of the genetic material and other accessible nucleic acids of the tumoral microbiome, can expose the microbiota to a heightened DNA damage stress and immune surveillance. Microbial response to these multipronged stresses on their genetic material is an important factor deserving attention, for an overall functional understanding of tumor associated microbial communities.

## Responding to the insults—microbial (counter) interactions

Microorganisms have evolved over billions of years to develop regulatory machineries for mitigating environmental stresses through well-orchestrated gene regulatory networks [62]. The stringent stress response and the general stress response are two key well-founded hallmarks of the stress regulatory responses in microbes [62, 63]. Depending upon the nature of stress ‘perceived’, as described in the subsequent sections, microbes can switch to an appropriate response mechanism for survival. Survival (and resilience) however is a function of ‘facilitation’ under a harsh environment and ‘persistence’ through the complex intra/interspecies interactions (e.g. competition and cooperation) [64]. This is also described by Chesson in the species co-existence theory, attributing a stabilized community structure to the influence of the environment on inter/intraspecies interactions including the consequent tolerance of invaders/stabilized community to the mutual competition [65, 66]. The competitive phenotypes of microbes broadly fall into two categories—(i) interference phenotypes and (ii) exploitative phenotypes [67, 68]. Interference competition occurs when the ability of a microbe to survive or attain resources is directly thwarted by interfering phenotypes or antagonistic interactions like chemical warfare and contact dependent-killing. Production of broad-spectrum antibiotics and strain-specific bacteriocins to eliminate rival microorganisms is a typical example of this chemical warfare mediated interference competition [67, 69]. Exploitative competition on the other hand is an indirect competition, experienced when microbes attempt to survive in a resource limited environment among competitors with overlapping nutrient requirements [67]. This entails phenotypes like secretion of nutrient-harvesting molecules (e.g. siderophores for iron sequestration), upregulation of transport or uptake pathways, secretion of digestive proteases, nucleases and even secretion of toxins like bacteriocins to specifically inhibit microorganisms with overlapping nutrient needs [63, 67, 68]. An insight into the competition sensing mechanisms in the microorganisms in fact rationally indicates that exploitative competition generates the interference competition between the microbes, with the larger goal of ruling out any contest for the resources by adopting strategies which can inhibit, displace, or kill the competitors [63]. As Cornforth and Foster propose, an umbrella term of “competition sensing” is less restrictive. It allows an emphasis on the ability of the microbes to sense any harmful stimulus or stressor, perceiving its origins in potential competitors, self or non-self [63]. The suitability and strength of the response to the perceived stimuli would therefore dictate the fate and function(s) of a microbial ecosystem. Given the heterogenous nature of tumor-microenvironment, the dynamics governing the multi-species stress response and competition under the harsh and variable environment of cancer [65, 66] potentially hold an important key to understand tumor-microbe interplay. Simply put, the balance of *‘the stress, the stress response and survival’* in the tumor micro(b)environment can govern the dynamics of crosstalk between *‘the cancer and the microbes’.* Notably though, despite the microbial stress response being defensive and compensative in nature [63, 67], it may not necessarily inhibit the cause of stress, i.e., cancer. This is unlike the response against competing microorganisms, where one microbe or community tries to win against the other (the world of microbe-kills-microbe)[63]. The composition of microbial community, density of the microbial populations, tumor physiology, the nature and the quantum of the evoked microbial stress response and the immunological response against microbial invasion is expected to decide the anti-tumor or tumorigenic role of the tumor microbiome.

For simplicity in describing the overarching theme of this article (environmental insults and compensative responses), bacterial ecology and stress response mechanisms will primarily be emphasized in the subsequent sections. Bacteria, the most abundant microorganisms inside human body, after all are prolifically studied, offering well founded and valuable models for understanding microbial response to environmental stresses. The terms ‘microbes and bacteria’ would therefore be used interchangeably.

### Doing collateral damage—Tumor targeting response of microbes

**Fig. 3:**
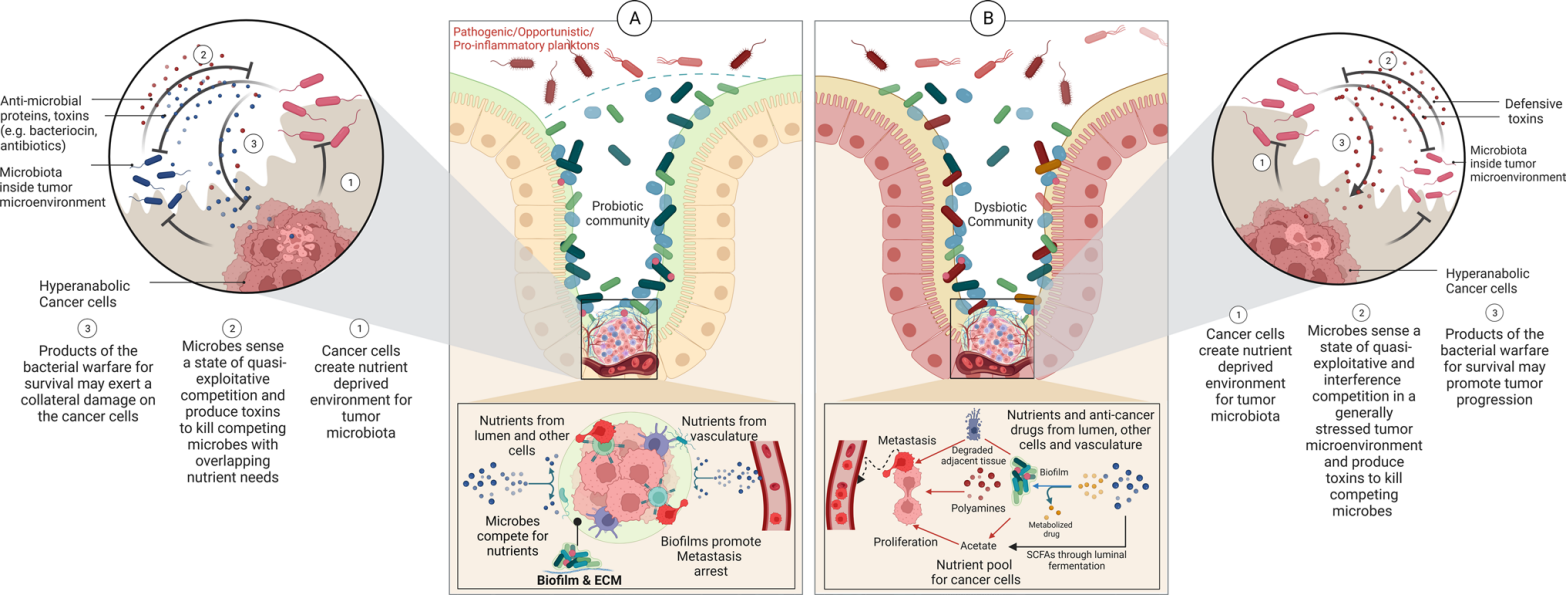
Microbial response to nutrient limited and stressed tumor microenvironment. **A** Microbes may inflict collateral damage on the tumor cells through competitive uptake of nutrients by the tumor microbiota. Expression of nutrient stress linked phenotype (e.g. Biofilms) can aggravate the nutrient stress on hyperanabolic tumor cells and can also prevent dispersion of cancer stem cells to other tissues (metastasis arrest). A quasi-exploitative competition between the microorganisms may also ensue, leading to production of antimicrobial peptides/toxins (e.g. bacteriocins) to thwart competition from microbes with overlapping nutrient needs. Prevalence of a probiotic microbial community in and around the tumor microenvironment is expected to cause more collateral damage to cancer than a (dysbiotic) community of pathobionts. **B** Biofilms inside the tumor microenvironment, produced in response to the nutrient stress can favor cancer proliferation and metastasis through polyamine biosynthesis, degradation of therapeutic drugs and disruption of normal adjacent tissue. Competition for survival between pathobionts (due to a dysbiotic native community) may lead to upregulation of pro-inflammatory microbial toxins, further promoting the cancer progression

The stringent stress response (SSR) is an evolutionary conserved specific stress response mechanism, mediated by the alarmone ‘guanosine tetraphosphate (ppGpp)’, that allows bacteria to reprogram their transcriptional activities when faced with nutrient stress (e.g. amino-acid, fatty acid and iron limitations) [70, 71]. This entails a switch from translation and biosynthesis to upregulated accumulation of limited resources [63, 70]. The state of nutrient stress offered by hyper anabolic cancer cells, aggravated by the overlapping nutrient requirements of the tumoral microbes, can evoke the SSR in the tumor-microbiota. This can reciprocate nutrient stress on cancer (Fig. 3), limiting its proliferation by competing for the nutrients critical for tumor progression, particularly branched chain amino acids (BCAA), acetate and iron [72–74]. Ecologically, a quasi-exploitative competition between the microbes sensing the competitive nutrient environment can elicit secretion of antimicrobial peptides/toxins like bacteriocins and other antibiotics. These microbiome derived molecules, primarily produced to fend off the perceived competition from the microbes with overlapping nutrient requirements, subject to the thriving of a favourable microbial community, may potentially inhibit the cancer cells in collateral damage (Fig. 3A) [75, 76]. A significantly high production of colicins and microcins (anti-cancer bacteriocins) by mucosal microbiome in CRC patients provides encouraging evidence in this regard [77]. The evidence pertaining to the ability of bacteriocins to cross epithelial and vascular endothelial cells add to the plausibility of a targeted response not only by the intra-tumoral microbes, but by the luminal, mucosal, NAT or stromal microbiome as well [78].

The presence of a global ‘General Stress Response (GSR)’ mechanism in bacteria, is however a key weapon in their arsenal of defence against a broad range of environmental insults [79]. It is mediated by the specialized transcriptional sigma (σ) factor(s) that compete with the house keeping sigma factor to redirect transcription towards hundreds of prokaryotic stress response genes, collectively called the general stress regulon. [79, 80]. Physio-biochemical stresses triggering the expression of this regulon are rather well founded. These include bacterial exposure to nutrient starvation, free radicals, heat, osmotic imbalance, acids, alcohols, membrane & DNA damaging environmental stimuli and more that (threaten to) compromise the integrity and survival of a microbial cell [80]. Given the association of GSR with a regulon consisting of hundreds of compensative genes, the phenotypic output of this defence mechanism is multi-pronged and confers a broad cross-resistance against a variety of rather unrelated stresses [79]. Accumulation of nutrients (e.g. glycogen, amino acids, acetate, iron, etc.), shift to fermentation and biofilm formation, expression of enzymes like catalases and oxidoreductases, accumulation or synthesis of osmoprotectants (e.g. trehalose, amino acids, K^+^), heightened expression of ‘amino acid decarboxylases, deaminases, proton pumping, biofilm formation’ for acid tolerance are few classical examples of GSR phenotypes [79–84]. It is also pertinent to note the association of GSR with transition to the stationary growth phase which is marked by a metabolic switch to the accumulation of inhibitory by-products/secondary metabolites like antibiotics, toxins and even complex behaviours like biofilm formation [79].

**Fig. 4:**
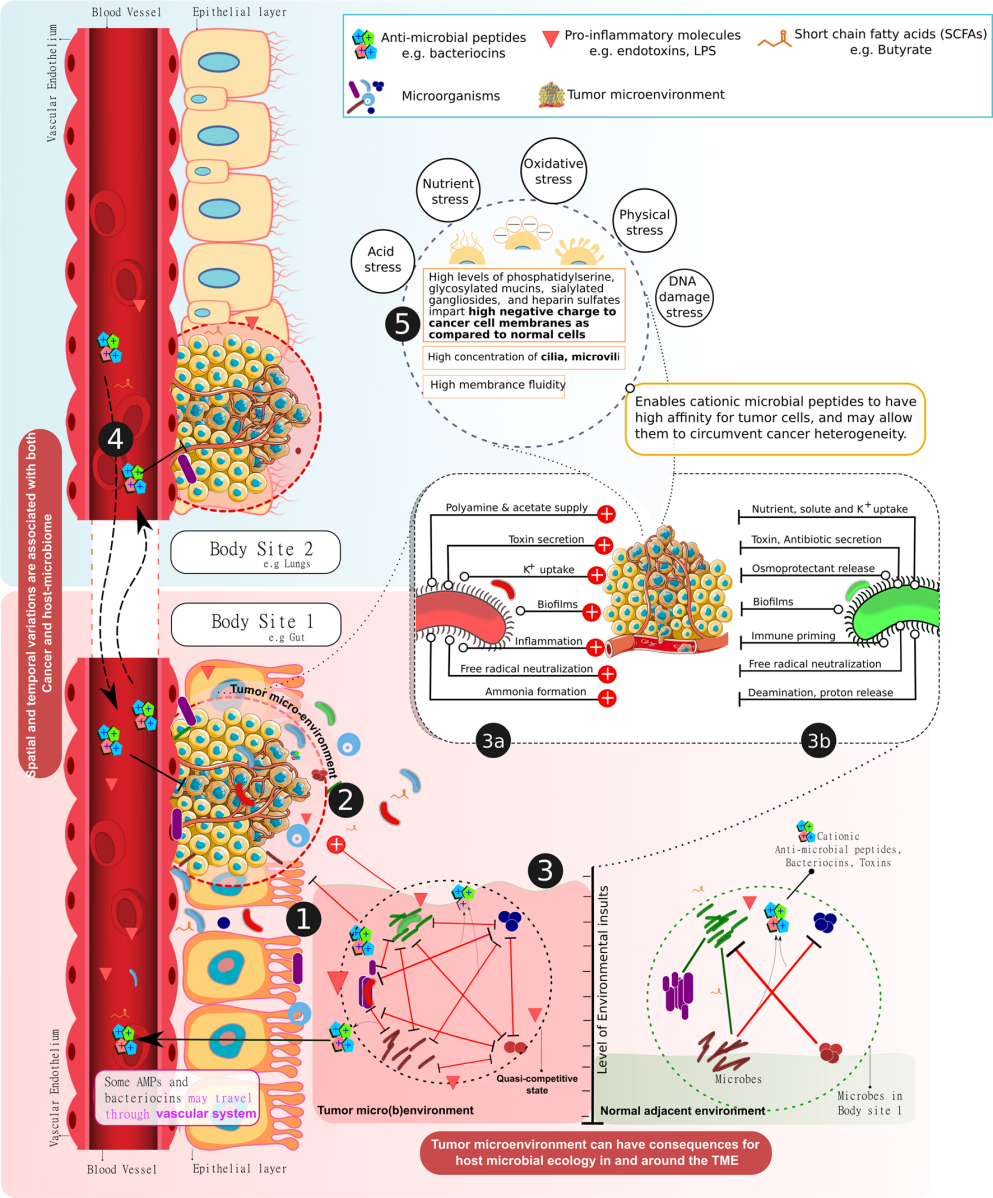
Microbial quest for survival in the tumor affected ecosystem. Microbes and microbial products may infiltrate to tumors through dysfunctional epithelial barrier, adjacent tissues or circulatory system. (2) Stressful tumor environment can trigger stringent and general stress response in microbes. (3) Environmental insults can lead to quasi-exploitative and quasi-interference competition between tumor microbes. (3a) Competitive environment and resultant stress response manifests in the form of upregulation of nutrient and ion uptake, synthesis of anti-microbial peptides/toxins, shift to fermentation, biofilm formation, redox balance and more causing collateral damage to cancer. (3b) Microbial responses can be oncogenic/promoting too (e.g. toxin secretion, polyamine metabolism, ammonia formation, inflammatory LPS, lowered oxidative stress on tumor, potassium influx). (4) Microbial metabolites including toxins and AMPs can access circulatory system for potential systemic effects. (5) Properties of cancer cell membranes can enable targeted attack by cationic anti-microbial peptides

The diverse environmental insults offered by tumor microenvironment to the invading/thriving microbes are expected to trigger the expression of the aforementioned general stress regulon. This is particularly true for nutrient and oxidative stress (abundant in the TME) which are known to confer a broad cross-protectivity through the activation of general stress response [80]. Table 1, backed by literature evidence, is compiled to describe the the key GSR linked phenotypic outcomes that can (potentially) inflict a collateral reciprocation of insults on the cancer cells. The relevant tumorigenic/tumor-promoting outcomes of the said GSR expression are summarised in the Fig. 4 and in the subsequent sections of this article.

**Table 1:** Potential responses of tumor invading/inhabiting microbiota mediated by the expression of GSR regulon under diverse environmental insults of the TME

GSR target	Phenotype	Mechanism of collateral damage for cancer	References
Nutrient stress	Accumulation of nutrients (e.g. glycogen, amino acids, acetate, iron, etc.) Shift to fermentation Biofilm formation	Resource limitation for hyperanabolic cancer cells (cancers need glycogen, acetate, iron, BCAA, etc.) Anti-mitotic role of SCFAs Metastasis distraction by biofilms through secretion of exopolysaccharides, preventing cancer cell binding to the endothelial cells	[81, 85–87]
Oxidative stress	Expression of free radical scavenging enzymes and molecules like catalases, oxidoreductases, Superoxide dismutase and mycothiol respectively Damage repairing proteins like thioredoxins, glutaredoxins, and methionine sulfoxide reductases	Free radical clearance and release of damage repairing proteins limits DNA damage, inflammatory cytokines, metastasis, and oncogenic mutagenesis	[5, 18, 88]
Acid Stress	Expression of amino acid (Arginine and Glutamate) decarboxylases activation of Arginine deaminase system Proton pumping Increased glycolytic activity Biofilm formation	Cancer cells are arginine addict (deprivation leads to cancer cell death) Glutamate is a key substrate for cancer cells Proton release by intra-tumor microbes can disrupt pH of cancer cells Heightened microbial glycolytic activity and biofilm formation can compete for energy metabolism and prevent metastasis	[39, 50, 89]
Physical Stress	Upregulated MSCRAMMs* and biofilm formation Expression of autolysin like enzymes, release of eDNA, teichoic acid and other cytoplasmic contents Upregulation of virulence factors like surface endonucleases	MSCRAMMs mediate covalent binding leading to persistent biofilms that can compete for nutrition and arrest metastasis eDNA and teichoic acids can mediate non-covalent binding of microbes to cancer, and can also trigger immune surveillance for collateral recognition of cancer cells Degradation of NETs and other entrapments by surface endonucleases can prevent metastasis	[47, 48, 53, 90, 91]
Osmotic stress	Solute uptake including amino acids, potassium ions (K^+^) Synthesis and accumulation of Trehalose	Uptake of amino acids as solutes can limit cancer energy metabolism Trehalose released through mechanosensitive channels and upon bacterial lysis can reduce inflammation, limit free radicals, enhance apoptosis Uptake of the storm of K^+^ ions released by dying cancer cells can limit suppression of cancer killing T-cell effector function	[72, 92, 93]
DNA damage	SOS response upregulates–biofilms with (drug resistant) persister population Intraspecies competition and consequent toxin secretion Toxin-anti toxin (TA) system activation Horizontal gene transfer	Persistent biofilms can compete for energy metabolism and prevent metastasis Toxins against intraspecies competition (e.g. colicins) can inhibit cancers TA systems can specifically cause cancer cell death (e.g. MazF-MazE toxin–antitoxin of E.coli against pancreatic and colorectal cancers) Anti-cancer toxins/antibiotics encoded by plasmids can promote population level phenotype through HGT	[63, 75, 94–97]

Notably, the GSR targets can be ameliorated by any stressor that can activate entire general stress regulon (conferring cross protectivity)

*MSCRAMM* Microbial Surface Components Recognizing Adhesive Matrix Molecules are microbial surface proteins that adhere specifically to host extra-cellular matrix (ECM), *eDNA* extracellular DNA, *NETs* Neutrophil Extracellular Traps

Under the right tumor microbial composition (native or interventional), this GSR and stationary phase linked in-vivo production of compensative products may even support cancer-therapy by priming the onco-immune system towards anti-tumor effects. The reported role of intra-tumoral probiotic gut-microbes in facilitating immunotherapy through the secondary metabolite mediated triggering of the STING signalling (stimulator of interferon genes), highlights this significance of tumoral colonization by commensal bacteria like *Bifidobacterium spp.* [98]*.*

From an ecological point of view, insults like oxidative stress, DNA damage stress, physical stress and acid stress are perceived as instances of direct challenges interfering with the ability of the microbes to survive and thrive. This calls for an activation of interference competitive phenotype and hence release of antibiotics and strain-specific bacteriocins towards the microbe-kill-microbe response [67]. The collateral damage inflicted on the cancer cells by this chemical warfare started by microbes under the perceived interference competition is plausible and therefore deserves exploration. The molecular mechanistic details underpinning this warfare may be described by the evolutionary matured stress response mechanisms as described earlier. In addition to the development of functional models, this would be important for the design of live biotherapeutics or dietary interventions aiming to favourably customize the microbial and metabolite composition of tumor invading/prevailing microbiota. Figure 4 provides a graphical overview of the (aforementioned) events that may ensue in tumor micro(b)environment.

### Into the wounds that never heal—tumor promoting response of microbes

While Rudolph Virchow first linked chronic inflammation with tumor development [99], Harold Dvorak’s comparison of tumors with the ‘Wounds that never heal’ notified similarities between tumor stroma generation (essential for tumor growth) and wound healing [44]. Microbial invasion of these wounds can spur the inflammation process [100], supporting the tumor elicited inflammation characterised by an accelerated recruitment of immune cells and up-regulation of pro-inflammatory cytokines and growth factors [100–103]. This can not only promote tumor progression but also aggravate the associated adverse symptoms. Notably, in-addition to the immune-regulating components of microbial anatomy like flagellin and lipopolysaccharide (LPS), the secondary metabolic products of microbial stress response like toxins (e.g. colibactin in certain strains of *Escherichia coli* and Bft toxin in *Bacteroides fragilis*) can be pro-inflammatory and oncogenic [15, 100, 101, 104]. These, as interjected earlier, are expected to be elicited in response to the diverse environmental insults faced by the invading microorganisms (Table 1).

The responses controlled by the general stress regulon may additionally support tumor progression (Fig. 4). This includes (i) the neutralization of oxidative stress by microbes in the tumor microenvironment, thereby lowering the compensative load on tumor cells which are also sensitive to redox imbalance [5, 88] (ii) acid stress management by microbial urease system leading to the formation of normally cytotoxic, proinflammatory but a potent nitrogen-reservoir for cancer cells - ammonia [84, 105] (iii) influx of potassium ions upon activation of osmotic stress response in the microbes, lowering intracellular tonicity of tumors and limiting T-cell stemness that enables cancer clearance [7, 106, 107] and (iv) the reported role of colonization assisting virulence factors (like FadA in *Fusobacterium nucleatum*) and stress resilient bacterial biofilms, a phenotypic response expected against nutrient, physical, DNA damage and acid stress, in initiation and progression of cancer (through LPS mediated inflammation, polyamine metabolism, toxin secretion and other well founded pro-oncogenic responses) is worth consideration as well (Fig. 3B) [108, 109]. Furthermore, the fermentative state of microbial growth under anoxic and nutrient depleted environment of tumors and normal adjacent tissue (e.g. gut epithelium and lumen) may contribute acetate (the most abundant SCFA), which, even though is reported for its anticancer potential, is also a key energy molecule for proliferating cancer cells (Fig. 3B) [73]. Noteworthy are the other well-founded microbial metabolites out of this fermentative state contributing to the pool of pathognomonic metabolites of cancer (oncometabolites) that accumulate in the TME due to genetic aberrations in the cancer cells, as well as infiltrating cancer associated fibroblasts and macrophages [110, 111]. These include fumarate, 2-hydroxyglutarate, succinate and lactate, that are known to aggravate cancer resilience, proliferation, hyper-anabolism, neoangiogenesis and aggressiveness [110, 111]. Consequently, this shared pool of oncometabolites among tumor and stressed microbial cells is expected to further spur the state of cancer progression.

Unsurprisingly, the molecular basis of ecological interactions of tumor prevailing/invading microbes with the potentially insulting environmental conditions dictate that the meeting of microbes with cancer can have both deleterious and advantageous consequences for the tumor. Where the balance would weigh more, can only be determined by the stabilized (or intervened) microbial population and its functional potential. It is therefore important, as we next discuss, to ponder over the directions that can branch out of this school of thought and potential limitations in assuming the native microbial populations of tumors, including any microbe-tumor cross-talk.

## Future directions

Human body essentially serves as an ecosystem to the colonizing microbes. The organ and tissue specific (spatio-temporal) territories of host microbiome are governed by the myriad of physiological, physical, metabolic and nutritional conditions specific to the sites of microbial colonization. Tumor development needs to be viewed as an ecological disturbance and its micro-environment as a perturbed niche capable of reshaping the structure of individual microbial populations through systemic and localized environmental pressures. How prevailing microbiota responds and survives against the ecological stresses offered by tumor development/progression is expected to drive the compositional and metabolic variations observed in different individuals, across different types of tumors. Such an understanding is critical to drive the development of in-silico models of tumor micro(b)environment through due attention to the dynamics of underlying metabolic fluxes and multi-species interactions (‘host-microbe, tumor-microbe, microbe-microbe and even tumor-tumor’). A functional gradation and classification of key microbial players (e.g. drivers, passengers) identified inside the tumor micro-environment may enable validation of the well founded driver-passenger models of various types of cancer [112, 113]. Furthermore, domain informed artificial intelligence through the integration of onco-immunological knowledge with the ecological gradients in the TME may help develop contextual (and perhaps more accurate) machine learnt models for cancer classification, prognosis, survival analysis and even personalized therapy [23, 114]. Interpretation of models that can accurately classify tumours of various types using this integrated knowledge may further help identify the key players (microbial, metabolic, immunological, etc.) driving the cancer-diversity [113, 115]. This may translate into comprehensive knowledge graphs, inter-omic networks and metagenome scale metabolic models of the TME [116, 117]. Agent-based models that have consistently been used for studying cancer evolution and simulated microbial ecosystems independently, may also leverage a recalibration through integration of the discussed aspects of onco-ecology with onco-immunology [118, 119]. Importantly, the aforementioned advances towards the functional understanding of microbial response to tumor micro-environment can aid development of therapeutic regimens aimed at modulating microbial populations and function thereof inside and around the cancer. This includes, but not limited to the dietary, probiotic and prebiotic formulations that can assist an accelerated reshaping of host and tumor microbiome towards an ‘anti-cancer’ community [77, 120, 121]. Such interventional modulations would be critical towards evoking desired quanta of microbial functional responses, that in their native state may not exert sufficient and sustained benefits. It would also be pertinent to project the ambitious possibility of preventive cocktails of pro/prebiotics that can sustain non-conducive states for cancer growth in healthy, at risk or early stage subjects, by leveraging the knowledge of competitive warfare in the tumor micro(b)environment.

## Limitations and considerations

Cancer however is a complex group of diseases characterised not only by abnormally dividing hyper-anabolic cells, unique micro-environment and location or site-specific manifestations but multifactorial confounders like specialized care and aggressive therapeutic regimens (e.g. chemotherapy, radiotherapy, etc.) [6, 121, 122]. This milieu of confounding factors can significantly impact the systemic as well as the localized host microbial ecology which may not overlap with the expected or characteristic response of microbes inside and in vicinity of a treatment-naive tumor environment.

### Spatio-temporal and model system variations

#### Site of cancer and microbial co-localization

Nejman and colleagues identified tissue specific distinct territories of tumor microbiota, highlighting the anatomical diversity in the microbial signatures of the cancers of different body sites [18]. For example, breast tumor microbiota was observed to be highly rich and diverse when compared to tumors of other body sites [18]. Previously, distinct (pathogenic) microbial species had already been associated with tumors of different body sites e.g. *Helicobacter pylori* (gastric cancer), *Salmonella typhi* (gall bladder cancer), *Chlamydophila pneumoniae* (lung cancer) and *Fusobacterium nucleatum* (colon cancer) [10, 14, 16, 17]. This site-specific diversity limits the ability to develop universal models of tumor-microbe interactions. Microbial cells can also potentially localize within (intracellular) or as frequently observed outside the (extracellular) cancer cells. A comprehensive pathological analysis of various tumor cores by Nejman *et al.*, in fact indicated a predominated intracellular localization of microbial 16S rRNA and LPS inside the cytoplasm of cancer cells, a discovery warranting further reproduction [18]. Heterogeneity in tumor-microbe cross-talk is only expected to be further aggravated by the gradients of stress that might be experienced by microbial cells while navigating the (extracellular) tumor microenvironment for any intra-cellular localization within the tumor and tumor-supporting cells.

#### Stage of cancer and microbial co-evolution

The quantum of environmental stresses in the tumor microenvironment are expected to depend on the stage or severity of the cancer as well. This in turn may dictate the shape of the community that can sustain and evolve at each stage of cancer. The cataloguing of lesional and non-lesional gut mucosal communities across different stages of colorectal tumorigenesis by Nakatsu and colleagues corroborates the same [122]. It was observed that structurally unique metacommunities are established at each stage of neoplasm progression, with enrichment of *Fusobacterium spp.* and *Bacteroides fragilis* in colorectal cancer [122]. A recent report highlighting high tumor-bacterial diversity in advanced stage papillary thyroid carcinoma (PTC) lesions, as compared to mild lesions adds to the corroboration [22]. Evidence in support of a significantly perturbed microbial community in cancer associated tissue across various tumor types, as compared to matched and unmatched healthy samples, are rather well reported [10, 22, 28, 122].

#### Cancer model and Inter-individual variability

Significant research on cancer treatment and diagnosis is carried out in natural or artificial (including in-vitro and computational) preclinical models [123]. Preclinical research is both a mandatory part of drug development/approval pipeline, as well as a key to comprehensive screening before translation can be attempted in human subjects [123]. The challenges in validating or recalibrating models across preclinical and clinical tumor microbiome research are therefore worth consideration as well [123]. Additionally, the personalized nature of host microbiome, governed by an individual’s personal spatio-temporal, race, geography, diet and lifestyle related dynamics adds to the complexity of factors that need to be accommodated for arriving at translatable models or personalized interventions [11, 12, 124]. The variations can further be complicated by the co-morbidities or other disorders or genetic predispositions that can systemically perturb the host microbiome [124].

#### Microniches in the tumor microenvironment (TME)

The TME is highly heterogeneous encompassing different cell types, extra-cellular matrix, necrotic regions, spatial gradients of free radicals, growth factors, cytokines and more [3, 9]. The regions of spatial and molecular heterogeneity constitute specialized microniches in the TME. Notable regions may be classified into (i) hypoxia microniche (containing proliferating cancer cells, stripped off oxygen supply) [125] (ii) necrotic microniche (containing dead cells and necrotic tissue) [125] (iii) acidic surface/synaptic microniche (representing the surface of the TME, the junction between normal adjacent tissue and tumor microenvironment) [6, 29] (iv) metastatic microniche (containing disseminated tumor cells with growth factors and immune evasion) [126] and cancer stem cell microniche (containing highly aggressive cancer stem cells) [41, 126]. It has been observed that microbes aren’t uniformly distributed in the TME and rather preferentially localize themselves in specific microniches. Members of *Clostridium*, *Bifidobacterium* and *Salmonella* for example prefer hypoxic and nutrient rich necrotic microniche suitable to their anaerobic nature [125]. A recent attempt to profile microbial tumor distribution in oral and colorectal cancers through spatial transcriptomics also revealed the localization of microbes (especially *Fusobacterium*) in distinct niches with progressing cancer and suppressed immune surveillance [32]. A uniform distribution of microbes in the TME can therefore not be assumed. Considerations for different microniches suitable for the physiology of the target or interventional microbiota may hold significance for meaningful functional modelling of the TME.

### Treatment regimen

The systemic implications of surgical (like Ostomy) and case-dependent dosages and durations of invasive therapeutic regimens like radiation or chemotherapy add to the associated complications of the disease and its ecosystem [121, 123, 124]. As described in Sect. 3.5, the therapeutic regimen like chemotherapy can exert excess DNA damage stress and disrupt the host microbiome (including the tumor microbiota) [58]. This disruptive impact can vary based on the duration and dosages of the therapy. A progressively reducing alpha diversity of tumor microbiome over the course of radiotherapy in HPV-associated oropharynx cancer was infact recently reported [28]. A general shift in microbial composition attributed to the perioperative procedures like antibiotic administration, mechanical bowel preparation and dietary restrictions can also add to the confounders needing accommodation in modelling the tumor associated ecosystem [127]. In the event of surgical removal of tumors, the post-operative host microbiome often requires interventional reshape, preferably with anti-cancer effects. For example, montmorillonite is advised to cancer patients to avoid toxic effects of antibiotics on the probiotic microbial communities [128]. Controlling the evolution of tumor associated microbiota (through antidotes) towards beneficial personalized communities would therefore be challenging but critical under the variable stresses of different treatment regimen [127–129].

### Lengthy workflow and contaminations

Worth consideration are the challenges associated with reproducing the results of microbiome studies (the reproducibility crisis), especially considering the compositionally sparse microbiota of tumor [18]. Given the extremely low microbial load of tumor associated samples, contaminants become an additional and key bottleneck to address against innumerable sources of contamination throughout the lengthy workflow of a microbiome study [18, 19].

## Conclusion

Surviving and thriving are key to organismal existence in the living world, microbes are no exception. Appreciating the challenges associated with colonization of an environment as complex and heterogeneous as the TME and linking them with what is well founded in microbial ecology can drive foundational understanding of microbial role in modulating the tumor microenvironment. Here an effort was made to characterize the relevant stresses in the tumor microenvironment that may serve as insults compromising the colonization and survival of microbes in the harsh environment of the tumors. Upon revisiting the classical evidence of microbial ecology/competition and stress response, it becomes encouragingly clear that collateral impact of microbial compensative responses to the consistent insults of the TME could hold an important key for developing functional models of tumor-microbe interaction. The success of various dietary regimens and microbial interventions (e.g. pre/probiotics), that attempt to channelize the host-microbial arsenal for cancer prevention or treatment may after all have roots in the basic concept of microbial competition sensing and their response to the environmental stimuli [63, 130, 131]. Understanding such stimuli in tumor micro(b)environment and microbial responses to the same, may therefore be critical to throw light on what happens (and can happen), when microbiota meets cancer.

## Abbreviations

TME: Tumor Microenvironment
NAT: Normal adjacent tissue
GSR: General Stress Response/Regulon
SSR: Stringent stress response
BCAA: Branched chain amino acids
SCFA: Short chain fatty acids
STING: Stimulator of interferon genes
LPS: Lipopolysaccharide

## References

[1] Hanahan D, Weinberg RA (2011). Hallmarks of cancer: the next generation. Cell.

[2] (US) NI of H, Study BSC. Understanding Cancer 2007

[3] Anderson NM, Simon MC (2020). The tumor microenvironment. Curr Biol.

[4] Hanahan D (2022). Hallmarks of cancer: new dimensions. Cancer Discov.

[5] Hayes JD, Dinkova-Kostova AT, Tew KD (2020). Oxidative stress in cancer. Cancer Cell.

[6] Boedtkjer E, Pedersen SF (2020). The acidic tumor microenvironment as a driver of cancer. Annu Rev Physiol.

[7] McGrail DJ, McAndrews KM, Brandenburg CP (2015). Osmotic regulation is required for cancer cell survival under solid stress. Biophys J.

[8] Dzobo K (2020). Taking a full snapshot of cancer biology: deciphering the tumor microenvironment for effective cancer therapy in the oncology clinic. OMICS.

[9] Xiao Y, Yu D (2021). Tumor microenvironment as a therapeutic target in cancer. Pharmacol Ther.

[10] Sepich-Poore GD, Zitvogel L, Straussman R (2021). The microbiome and human cancer. Science.

[11] Ursell LK, Metcalf JL, Parfrey LW, Knight R (2012). Defining the human microbiome. Nutr Rev.

[12] Baquero F, Nombela C (2012). The microbiome as a human organ. Clin Microbiol Infect.

[13] Coley WB (1910). The treatment of inoperable sarcoma by bacterial toxins (the mixed toxins of the *Streptococcus erysipelas* and the *Bacillus prodigiosus*). Proc R Soc Med.

[14] Parida S, Sharma D (2021). The microbiome and cancer: Creating friendly neighborhoods and removing the foes with in A C. Cancer Res.

[15] Wong-Rolle A, Wei HK, Zhao C, Jin C (2021). Unexpected guests in the tumor microenvironment: microbiome in cancer. Protein Cell.

[16] Mager DL (2006). Bacteria and cancer: cause, coincidence or cure? A review. J Transl Med.

[17] Parsonnet J (1995). Bacterial infection as a cause of cancer. Environ Health Perspect.

[18] Nejman D, Livyatan I, Fuks G (2020). The human tumor microbiome is composed of tumor type-specific intracellular bacteria. Science (1979).

[19] Robinson KM, Crabtree J, Mattick JSA (2017). Distinguishing potential bacteria-tumor associations from contamination in a secondary data analysis of public cancer genome sequence data. Microbiome.

[20] Atreya CE, Turnbaugh PJ (1979). Probing the tumor micro(b)environment. Science (1979).

[21] Choi JK, Naffouje SA, Goto M (2023). Cross-talk between cancer and *Pseudomonas aeruginosa* mediates tumor suppression. Commun Biol.

[22] Yuan L, Yang P, Wei G (2022). Tumor microbiome diversity influences papillary thyroid cancer invasion. Commun Biol.

[23] Hermida LC, Gertz EM, Ruppin E (2021). Analyzing the tumor microbiome to predict cancer patient survival and drug response. Cancer Res.

[24] Feng Z, Hu Y, Wang X (2022). In situ imaging for tumor microbiome interactions via imaging mass cytometry on single-cell level. Cytometry A.

[25] Thyagarajan S, Zhang Y, Thapa S (2020). Comparative analysis of racial differences in breast tumor microbiome. Sci Rep.

[26] Murphy CL, Barrett M, Pellanda P (2021). Mapping the colorectal tumor microbiota. Gut Microbes.

[27] Okuda S, Shimada Y, Tajima Y (2021). Profiling of host genetic alterations and intra-tumor microbiomes in colorectal cancer. Comput Struct Biotechnol J.

[28] Bahig H, Fuller CD, Mitra A (2021). Longitudinal characterization of the tumoral microbiome during radiotherapy in HPV-associated oropharynx cancer. Clin Transl Radiat Oncol.

[29] Livyatan I, Nejman D, Shental N, Straussman R (2020). Characterization of the human tumor microbiome reveals tumor-type specific intra-cellular bacteria. Oncoimmunology.

[30] Guo W, Zhang Y, Guo S (2021). Tumor microbiome contributes to an aggressive phenotype in the basal-like subtype of pancreatic cancer. Commun Biol.

[31] Zwinsová B, Petrov VA, Hrivňáková M (2021). Colorectal tumour mucosa microbiome is enriched in oral pathogens and defines three subtypes that correlate with markers of tumour progression. Cancers (Basel).

[32] Niño JLG, Wu H, LaCourse KD (2022). Effect of the intratumoral microbiota on spatial and cellular heterogeneity in cancer. Nature.

[33] Poore GD, Kopylova E, Zhu Q (2020). Microbiome analyses of blood and tissues suggest cancer diagnostic approach. Nature.

[34] Mullin JM (2004). Epithelial barriers, compartmentation, and cancer. Sci STKE.

[35] Soler AP, Miller RD, Laughlin KV (1999). Increased tight junctional permeability is associated with the development of colon cancer. Carcinogenesis.

[36] Cróinín O, T, Backert S, (2012). Host epithelial cell invasion by *Campylobacter jejuni*: trigger or zipper mechanism?. Front Cell Infect Microbiol.

[37] Cummins J, Tangney M (2013). Bacteria and tumours: causative agents or opportunistic inhabitants?. Infect Agent Cancer.

[38] Hashizume H, Baluk P, Morikawa S (2000). Openings between defective endothelial cells explain tumor vessel leakiness. Am J Pathol.

[39] Sullivan MR, Vander Heiden MG (2019). Determinants of nutrient limitation in cancer. Crit Rev Biochem Mol Biol.

[40] Liberti MV, Locasale JW (2016). The warburg effect: how does it benefit cancer cells?. Trends Biochem Sci.

[41] Yadav UP, Singh T, Kumar P (2020). Metabolic adaptations in cancer stem cells. Front Oncol.

[42] Zhou S, Gravekamp C, Bermudes D, Liu K (2018). Tumour-targeting bacteria engineered to fight cancer. Nat Rev Cancer.

[43] Valko M, Leibfritz D, Moncol J (2007). Free radicals and antioxidants in normal physiological functions and human disease. Int J Biochem Cell Biol.

[44] Dvorak HF (2015). Tumors: wounds that do not heal-redux. Cancer Immunol Res.

[45] Flier JS, Underhill LH, Dvorak HF (1986). Tumors: wounds that do not heal. N Engl J Med.

[46] De Meo ML, Spicer JD (2021). The role of neutrophil extracellular traps in cancer progression and metastasis. Semin Immunol.

[47] Brinkmann V, Reichard U, Goosmann C (2004). Neutrophil extracellular traps kill bacteria. Science (1979).

[48] Yang L, Liu Q, Zhang X (2020). DNA of neutrophil extracellular traps promotes cancer metastasis via CCDC25. Nature.

[49] Masucci MT, Minopoli M, Del Vecchio S, Carriero MV (2020). The emerging role of neutrophil extracellular traps (NETs) in tumor progression and metastasis. Front Immunol.

[50] Lee SH, Griffiths JR (2020). How and why are cancers acidic? Carbonic anhydrase ix and the homeostatic control of tumour extracellular ph. Cancers (Basel)..

[51] Evans DF, Pye G, Bramley R (1988). Measurement of gastrointestinal pH profiles in normal ambulant human subjects. Gut.

[52] Fischer H, Widdicombe JH (2006). Mechanisms of acid and base secretion by the airway epithelium. J Membrane Biol.

[53] Otto M (2014). Physical stress and bacterial colonization. FEMS Microbiol Rev.

[54] Maurice CF, Haiser HJ, Turnbaugh PJ (2013). Xenobiotics shape the physiology and gene expression of the active human gut microbiome. Cell.

[55] Johnson NP, Razaka H, Wimmer F (1987). Toxicity, mutagenicity and drug resistance in *Escherichia coli* treated with platinum antitumor compounds. Inorganica Chim Acta.

[56] Shapiro RS (2015). Antimicrobial-induced DNA damage and genomic instability in microbial pathogens. PLoS Pathog.

[57] Maier L, Pruteanu M, Kuhn M (2018). Extensive impact of non-antibiotic drugs on human gut bacteria. Nature.

[58] Papanicolas LE, Sims SK, Taylor SL (2021). Conventional myelosuppressive chemotherapy for non-haematological malignancy disrupts the intestinal microbiome. BMC Cancer.

[59] Balian A, Hernandez FJ (2021). Nucleases as molecular targets for cancer diagnosis. Biomark Res.

[60] Nagi RS, Bhat AS, Kumar H (2014). Cancer: a tale of aberrant PRR response. Front Immunol.

[61] Yang W (2011). Nucleases: diversity of structure, function and mechanism. Q Rev Biophys.

[62] Foster PL (2005). Stress responses and genetic variation in bacteria mutation research—fundamental and molecular. Mech Mutagen.

[63] Cornforth DM, Foster KR (2013). Competition sensing: the social side of bacterial stress responses. Nat Rev Microbiol.

[64] Hart SP, Marshall DJ (2013). Environmental stress, facilitation, competition, and coexistence. Ecology.

[65] Chesson P (1994). Multispecies competition in variable environments. Theor Popul Biol.

[66] Chesson P (2018). Updates on mechanisms of maintenance of species diversity. J Ecol.

[67] Hibbing ME, Fuqua C, Parsek MR, Peterson SB (2010). Bacterial competition: surviving and thriving in the microbial jungle. Nat Rev Microbiol.

[68] Ghoul M, Mitri S (2016). The ecology and evolution of microbial competition. Trends Microbiol.

[69] Bauer MA, Kainz K, Carmona-Gutierrez D, Madeo F (2018). Microbial wars: competition in ecological niches and within the microbiome. Microbial Cell.

[70] Irving SE, Choudhury NR, Corrigan RM (2021). The stringent response and physiological roles of (pp)pGpp in bacteria. Nat Rev Microbiol.

[71] Boutte CC, Crosson S (2013). Bacterial lifestyle shapes stringent response activation. Trends Microbiol.

[72] Sivanand S, Vander Heiden MG (2020). Emerging Roles for Branched-Chain Amino Acid Metabolism in Cancer. Cancer Cell.

[73] Schug ZT, Vande Voorde J, Gottlieb E (2016). The metabolic fate of acetate in cancer. Nat Rev Cancer.

[74] Manz DH, Blanchette NL, Paul BT (2016). Iron and cancer: recent insights. Ann N Y Acad Sci.

[75] Karpiński TM, Adamczak A (2018). Anticancer activity of bacterial proteins and peptides. Pharmaceutics.

[76] Dobson A, Cotter PD, Paul Ross R, Hill C (2012). Bacteriocin production: a probiotic trait?. Appl Environ Microbiol.

[77] Kohoutova D, Forstlova M, Moravkova P (2020). Bacteriocin production by mucosal bacteria in current and previous colorectal neoplasia. BMC Cancer.

[78] Dreyer L, Smith C, Deane SM (2019). Migration of bacteriocins across gastrointestinal epithelial and vascular endothelial cells, as determined using in vitro simulations. Sci Rep.

[79] Gottesman S (2019). Trouble is coming: signaling pathways that regulate general stress responses in bacteria. J Biol Chem.

[80] Boor KJ (2006). Bacterial stress responses: What doesn’t kill them can make them stronger. PLoS Biol.

[81] Dauer P, Lengyel E (2019). New roles for glycogen in tumor progression. Trends Cancer.

[82] Gottschlich L, Geiser P, Bortfeld-Miller M (2019). Complex general stress response regulation in Sphingomonas melonis Fr1 revealed by transcriptional analyses. Sci Rep.

[83] Bearson S, Bearson B, Foster JW (1997). Acid stress responses in enterobacteria. FEMS Microbiol Lett.

[84] Guan N, Liu L (2020). Microbial response to acid stress: mechanisms and applications. Appl Microbiol Biotechnol.

[85] Deberardinis RJ, Chandel NS (2016). Fundamentals of cancer metabolism introduction and overarching principles. Adv Sci.

[86] Ohara T, Mori T (2019). Antiproliferative effects of short-chain fatty acids on human colorectal cancer cells via gene expression inhibition. Anticancer Res.

[87] Sieow BFL, Wun KS, Yong WP (2021). Tweak to treat: reprograming bacteria for cancer treatment. Trends Cancer.

[88] Ezraty B, Gennaris A, Barras F, Collet JF (2017). Oxidative stress, protein damage and repair in bacteria. Nat Rev Microbiol.

[89] Al-Koussa H, El Mais N, Maalouf H (2020). Arginine deprivation: a potential therapeutic for cancer cell metastasis? A review. Cancer Cell Int.

[90] Beiter K, Wartha F, Albiger B (2006). An endonuclease allows *Streptococcus pneumoniae* to escape from neutrophil extracellular traps. Curr Biol.

[91] Ho SS, Michalek SM, Nahm MH (2008). Lipoteichoic acid is important in innate immune responses to gram-positive bacteria. Infect Immun.

[92] S N Chaitanya N, Devi A, Sahu S, Alugoju P, (2021). Molecular mechanisms of action of Trehalose in cancer: a comprehensive review. Life Sci.

[93] Eil R, Vodnala SK, Clever D (2016). Ionic immune suppression within the tumour microenvironment limits T cell effector function. Nature.

[94] Žgur-Bertok D (2013). DNA damage repair and bacterial pathogens. PLoS Pathog.

[95] Podlesek Z, Žgur Bertok D (2020). The DNA damage inducible SOS response is a key player in the generation of bacterial persister cells and population wide tolerance. Front Microbiol.

[96] Inglis RF, Bayramoglu B, Gillor O, Ackermann M (2013). The role of bacteriocins as selfish genetic elements. Biol Lett.

[97] Shapira S, Boustanai I, Kazanov D (2021). Innovative dual system approach for selective eradication of cancer cells using viral-based delivery of natural bacterial toxin–antitoxin system. Oncogene.

[98] Shi Y, Zheng W, Yang K (2020). Intratumoral accumulation of gut microbiota facilitates CD47-based immunotherapy via STING signaling. J Exp Med.

[99] David H (1988). Rudolf virchow and modern aspects of tumor pathology. Pathol Res Pract.

[100] Grivennikov SI, Wang K, Mucida D (2012). Adenoma-linked barrier defects and microbial products drive IL-23/IL-17-mediated tumour growth. Nature.

[101] Yang W, Cong Y (2021). Gut microbiota-derived metabolites in the regulation of host immune responses and immune-related inflammatory diseases. Cell Mol Immunol.

[102] Wang K, Karin M. Tumor-elicited inflammation and colorectal cancer. In: Advances in cancer research. 2015. 10.1016/bs.acr.2015.04.014 10.1016/bs.acr.2015.04.014 26216633

[103] Hou J, Karin M, Sun B (2021). Targeting cancer-promoting inflammation—have anti-inflammatory therapies come of age?. Nat Rev Clin Oncol.

[104] Shalapour S, Karin M (2020). Cruel to be kind: epithelial, microbial, and immune cell interactions in gastrointestinal cancers. Annu Rev Immunol.

[105] Li X, Zhu H, Sun W (2021). Role of glutamine and its metabolite ammonia in crosstalk of cancer-associated fibroblasts and cancer cells. Cancer Cell Int.

[106] Vodnala SK, Eil R, Kishton RJ (2019). T cell stemness and dysfunction in tumors are triggered by a common mechanism. Science (1979).

[107] Csonka LN (1989). Physiological and genetic responses of bacteria to osmotic stress. Microbiol Rev.

[108] Li S, Konstantinov SR, Smits R, Peppelenbosch MP (2017). Bacterial biofilms in colorectal cancer initiation and progression. Trends Mol Med.

[109] Wang N, Fang JY (2023). Fusobacterium nucleatum, a key pathogenic factor and microbial biomarker for colorectal cancer. Trends Microbiol.

[110] Baryła M, Semeniuk-Wojtaś A, Róg L (2022). Oncometabolites—a link between cancer cells and tumor microenvironment. Biology (Basel).

[111] Liu Y, Yang C (2021). Oncometabolites in cancer: current understanding and challenges. Cancer Res.

[112] Garza DR, Taddese R, Wirbel J (2020). Metabolic models predict bacterial passengers in colorectal cancer. Cancer Metab.

[113] Geng J, Song Q, Tang X (2014). Co-occurrence of driver and passenger bacteria in human colorectal cancer. Gut Pathog.

[114] Deng C, Ji X, Rainey C (2020). Integrating machine learning with human knowledge. IScience.

[115] Withnell E, Zhang X, Sun K, Guo Y (2021). XOmiVAE: an interpretable deep learning model for cancer classification using high-dimensional omics data. Brief Bioinform.

[116] Fu C, Zhong R, Jiang X, et al (2020) An integrated knowledge graph for microbe-disease associations. In: lecture notes in computer science (including subseries Lecture Notes in Artificial Intelligence and Lecture Notes in Bioinformatics). 10.1007/978-3-030-61951-0_8

[117] Zorrilla F, Buric F, Patil KR, Zelezniak A (2021). MetaGEM: reconstruction of genome scale metabolic models directly from metagenomes. Nucleic Acids Res.

[118] Shashkova T, Popenko A, Tyakht A (2016). Agent based modeling of human gut microbiome interactions and perturbations. PLoS ONE.

[119] Wang Z, Butner JD, Kerketta R (2015). Simulating cancer growth with multiscale agent-based modeling. Semin Cancer Biol.

[120] Hols P, Ledesma-García L, Gabant P, Mignolet J (2019). Mobilization of microbiota commensals and their bacteriocins for therapeutics. Trends Microbiol.

[121] Chakrabarty AM (2003). Microorganisms and cancer: quest for a therapy. J Bacteriol.

[122] Nakatsu G, Li X, Zhou H (2015). Gut mucosal microbiome across stages of colorectal carcinogenesis. Nat Commun.

[123] Sajjad H, Imtiaz S, Noor T (2021). Cancer models in preclinical research: a chronicle review of advancement in effective cancer research. Animal Model Exp Med.

[124] Vujkovic-Cvijin I, Sklar J, Jiang L (2020). Host variables confound gut microbiota studies of human disease. Nature.

[125] Song S, Vuai MS, Zhong M (2018). The role of bacteria in cancer therapy—enemies in the past, but allies at present. Infect Agent Cancer.

[126] Celià-Terrassa T, Kang Y (2018). Metastatic niche functions and therapeutic opportunities. Nat Cell Biol.

[127] Koliarakis I, Athanasakis E, Sgantzos M (2020). Intestinal microbiota in colorectal cancer surgery. Cancers (Basel).

[128] Rao Malla R, Marni R, Kumari S (2022). Microbiome assisted tumor microenvironment: emerging target of breast cancer. Clin Breast Cancer.

[129] Park DS, Robertson-Tessi M, Luddy KA (2019). The goldilocks window of personalized chemotherapy: getting the immune response just right. Cancer Res.

[130] Kolodziejczyk AA, Zheng D, Elinav E (2019). Diet–microbiota interactions and personalized nutrition. Nat Rev Microbiol.

[131] Lee C, Longo VD (2011). Fasting vs dietary restriction in cellular protection and cancer treatment: from model organisms to patients. Oncogene.

